# Cerebrovascular events and thrombolysis in pulmonary embolism-induced cardiac arrest: a case series and key challenges

**DOI:** 10.1186/s12872-024-04012-9

**Published:** 2024-07-27

**Authors:** Youping Zhang, Shu Peng, Karl Nelson S.Marquez, Xiangning Fu, Bo Ai, Hua Yan, Wei Zhu, Shusheng Li

**Affiliations:** 1 Department of Emergency Medicine, Tongji Hospital, Tongji Medical College, Huazhong University of Science and Technology, 1095 Jiefang Ave, Wuhan, 430030 Hubei P.R. China; 2 Department of Critical Care Medicine, Tongji Hospital, Tongji Medical College, Huazhong University of Science and Technology, 1095 Jiefang Ave, Wuhan, 430030 Hubei P.R. China; 3 Department of Thoracic Surgery, Tongji Hospital, Tongji Medical College, Huazhong University of Science and Technology, Wuhan, Hubei P.R. China; 4 Clinical Medicine, Tongji Medical College, Huazhong University of Science and Technology, Wuhan, Hubei P.R. China

**Keywords:** Cerebrovascular events, Cardiac arrest, Cardiac pulmonary resuscitation, Pulmonary embolism, Thrombolysis, Case series

## Abstract

**Background and purpose:**

Cerebrovascular events during thrombolysis in cardiac arrest (CA) caused by pulmonary embolism (PE) is a life-threatening condition. However, the balance between cerebrovascular events and thrombolytic therapy in PE-induced CA remains a great challenge.

**Methods:**

In this study, we reported three unique cases regarding main concerns surrounding cerebrovascular events in thrombolytic therapy in PE-induced CA.

**Results:**

The patient in the case 1 treated with thrombolysis during CPR and finally discharged neurologically intact. The patient in the case 2 received delayed thrombolysis and died eventually. The patient in the case 3 was contraindicated to thrombolysis due to the complication of subarachioid hemorrahage and died within days.

**Conclusions:**

Our case series highlights three proposed approaches to consider before administering thrombolysis as a treatment option in PE-induced CA patients: (1) **prolonging the resuscitation**, (2) **administering thrombolysis promptly**, and (3) **ruling out cerebrovascular events.**

## Background

Cerebrovascular events can be life-threatening if not treated accordingly. Thrombolysis is an emergency treatment option that can restore adequate circulation while performing cardiac pulmonary resuscitation (CPR) in cardiac arrest (CA) caused by pulmonary embolism (PE). Acute pulmonary embolism (PE) is one of the most common causes of cardiac arrest. It is estimated that PE contributes to approximately 10% of unexpected cardiac arrests with mortality rates ranging from 65 to 88% [1, 2]. Thrombolysis has emerged as one of the primary therapeutic approaches to rapidly reverse pulmonary artery occlusion and restore adequate circulation. In fact, thrombolytic therapy in PE-induced CA is a double-edged sword. Thrombolytic therapy can re-establish circulation but it can also lead to fatal haemorrhage. Thus, cerebrovascular events are important issues regarding thrombolysis in CA caused by PE. However, the benefits and risks of thrombolysis during CPR among patients with CA caused by PE remain a topic of debate. On the one hand, diagnosing PE during an intra-arrest situation is challenging. Hence, the current guidelines only recommend considering thrombolysis when the return of spontaneous circulation (ROSC) is not achieved, particularly when there is high suspicion of PE [3]. On the other hand, the balance between cerebrovascular events and thrombolytic therapy remains a great challenge. To shed light on the key concerns regarding cerebrovascular events in thrombolysis during CA caused by PE, this study presents three illustrative cases (Table 1).

## Case presentation

### Case 1

**Fig. 1:**
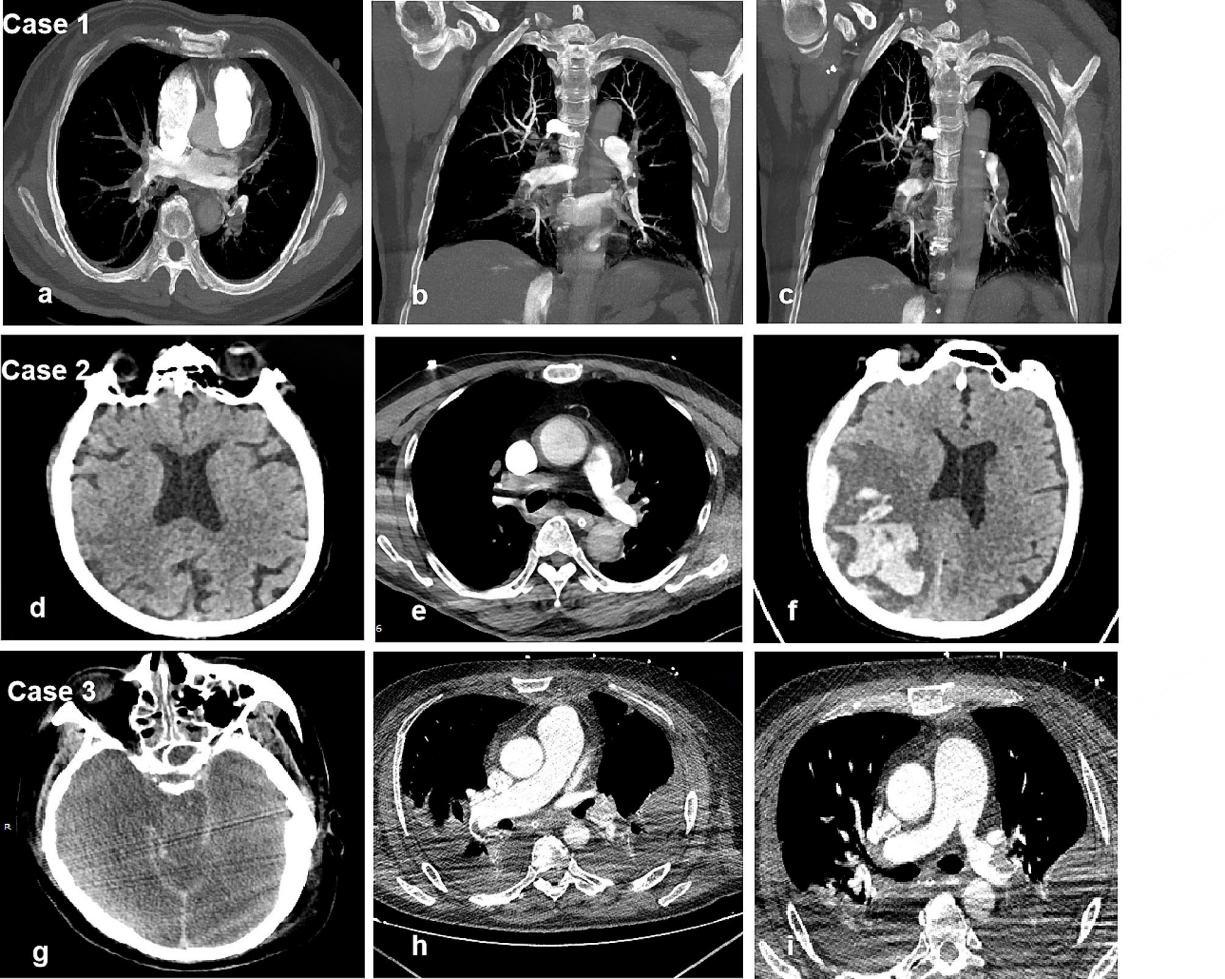
Images of 3 cases. Case 1 (**a-c**): Computed tomography pulmonary angiogram (CTPA) showed a bilaterally massive pulmonary embolism. Case 2: **d**, brain CT before thrombolysis was normal. **e**, CTPA revealed a massive pulmonary embolism. **f**, brain CT after thrombolysis showed hemorrhagic transformation following cerebral infarction. Case 3 : **g**, the brain CT revealed subarachnoid haemorrhage. **h-i**, CTPA showed massive left pulmonary artery embolism.

A 64-year-old woman developed dyspnea and syncope eight days after undergoing laparoscopic radical hysterectomy, double adnexectomy with aortic and pelvic lymphadenectomy. When the doctors arrived at the post-operative ward, she had a central pulse and was conscious but non-communicative. She remained haemodynamically unstable. Cardiac arrest occurred on her way back from computed tomography pulmonary angiogram (CTPA) which showed a bilaterally massive pulmonary embolism (Fig. 1: a-c). CPR was started and thrombolysis was discussed. Within five minutes, she was intubated and connected to a ventilator. Intravenous (IV) alteplase (5 mg) was administered five minutes later, followed by a 45 mg IV alteplase infusion within one hour. ROSC was obtained after 20 min, although not sustained. After 74 min of CPR, she finally achieved haemodynamic stability. Peripheral oxygen saturation increased from 60% (Fi02 100%) to 100% (Fi02 40%), and awareness returned three hours post-thrombolysis. Transient haematuria resolved completely within 24 h. She was discharged eight days later. A 30-day post-discharge neurological exam showed no neurological sequelae associated with prolonged CPR.

## Case 2

A 75-year-old man was hospitalized due to haematuria 10 days after right ureteral stent implantation. On the third day after transurethral cystectomy, he developed a sudden cardiac arrest while walking in the ward. Immediate intubation and bedside resuscitation were executed. After 22 min of CPR, ROSC was achieved, but the circulation remained unstable. Two hours later, radiographic examinations were conducted. Brain computed tomography (CT) scan was normal (Fig. 1: d), whereas CTPA revealed a massive pulmonary embolism (Fig. 1: e). An IV administration of 50 mg recombinant tissue-type plasminogen activator (rtPA) was initiated within five hours. Despite thrombolytic treatment, the patient remained in a deep coma, with later confirmation of hemorrhagic transformation following cerebral infarction via brain CT scan (Fig. 1: f). On the 5th day post-operative, his condition progressively deteriorated, leading to his death upon the family’s request to withdraw treatment (allowed by local legislation).

## Case 3

A 55-year-old man with fall related injury suddenly developed central cyanosis, Kussmaul breathing, and loss of consciousness while rising from his post-operative ward bed. Four days earlier, he underwent a spinal fracture fixation operation due to a first lumbar vertebra fracture and corresponding spinal cord contusion. Within minutes, he had a pulseless electrical activity cardiac arrest in the trauma surgery ward. CPR was initiated. He developed two episodes of ventricular fibrillation, which quickly resolved with defibrillation. He was intubated and connected to a ventilator. After 15 min of CPR, ROSC was obtained and he was transferred to the ICU. Pulmonary embolism is strongly suspected as major bleeding, pneumothorax, electrolyte imbalances, and airway obstruction were ruled out by blood gas analysis, echocardiography, and chest X-rays. However, brain CT or CTPA examination was not able to perform after the CA as he was too unstable for transport to the department of radiology. Prophylactic anticoagulation was not given due to concerns for complications of bleeding. On the second day after being transferred, hypoxemia and CA reoccurred without prior symptoms. Immediate CPR restored ROSC after 50 min. Despite re-established systemic circulation, he exhibited neurological abnormalities (pupillary light reflex -, diameter 4 mm). His fasting blood sugar level was normal. The brain CT revealed subarachnoid haemorrhage (Fig. 1: g) while CTPA showed massive left pulmonary artery embolism (Fig. 1: h-i). Due to bleeding risks, neither thrombolytic therapy nor anticoagulation was administered. On the 15th day in ICU, he died after family-requested treatment withdrawal (allowed by local legislation).

**Table 1:** Cerebrovascular events and thrombolysis in PE-induced CA patients CA, Cardiac Arrest; CPR, Cardiac Pulmonary Resuscitation; CT, Computed Tomography; CTPA, Computed Tomography Pulmonary Angiogram; PE, Pulmonary Embolism; ROSC, Return of Spontaneous Circulation; rtPA, Recombinant Tissue-type Plasminogen Activator.

	Case 1	Case 2	Case 3
Age (years)	64	75	55
Sex	Female	Male	Male
Chief complaint	Dyspnea, syncope	Sudden cardiac arrest during walking	Central cyanosis, Kussmaul breathing, loss of consciousness
Past interventions before cardiac arrest	8 days after laparoscopic radical hysterectomy, double adnexectomy, aortic, and pelvic lymphadenectomy	10 days after right ureteral stent implantation, 3 days after transurethral cystectomy	4 days after spinal fracture, 1st lumbar vertebra fracture, spinal cord contusion
Prophylactic anticoagulation before CA	low-molecular-weight heparin 3 days after the surgery (2125U QD)	No	No
Time to Intubation and Mechanical Ventilation	Within 5 min of CPR	Immediately after cardiac arrest	Immediately after cardiac arrest
Times of CA	1	1	2
ROSC / Haemodynamically Stable Reached	Haemodynamically stable after 74 min of CPR	ROSC reached after 22 min of CPR	1st CA: 15 min of CPR (ROSC) 2nd CA: 50 min of CPR (ROSC)
Imaging signs	Bilaterally massive pulmonary embolism (CTPA)	Normal brain CT, massive pulmonary embolism (CTPA)	Subarachnoid hemorrhage (Brain CT), massive pulmonary embolism (CTPA)
Massive PE detected	CTPA showed bilaterally massive pulmonary embolism	CTPA revealed massive pulmonary embolism	CTPA showed massive embolism of the left pulmonary artery
Bedside echocardiography	Normal (after CA)	Enlargement of the right ventricle (after CA)	Enlargement of the right ventricle (after CA)
Bedside vascular ultrasound	Deep vein thrombosis	Intermuscular vein thrombosis	Intermuscular vein thrombosis
Thrombolysis Dose	IV alteplase (5 mg) injection 5 min after CPR started, IV infusion (45 mg) 10 min after CPR started	IV rtPA (50 mg) 5 h after ROSC	Not given
Bleeding Complications	Transient hematuria, recovered in 24 h	Hemorrhagic transformation after cerebral infarction	Subarachnoid hemorrhage
Outcomes/ Other Complications	Peripheral oxygen saturation increased	Deep coma	Neurological abnormalities, ROSC achieved but cardiac arrest reoccurred
Result	Discharged after 8 days, fully recovered without neurological deficit	Died after treatment withdrawal, 5 days post-operative	Died after treatment withdrawal, 15 days post-operative

## Discussion and conclusions

Thrombolytic therapy in PE-induced CA is a double-edged sword. According to international guidelines [4], the decision to treat PE-induced through thrombolysis intra-arrest should be made with no delays. When contraindications to thrombolysis are identified, clinicians should still consider the possibility of administering thrombolytic treatment while considering the risks involved [5]. Our case series highlights three key challenges before administering thrombolysis in PE-induced CA patients: (1) prolonging the resuscitation, (2) administering thrombolysis promptly, and (3) ruling out cerebrovascular events.

## Prolonged resuscitation

In the first case, continuous CPR was diligently performed until the patient attained hemodynamic stability after 74 min of resuscitation efforts. This case highlights the suggested appropriate length time of CPR when thrombolysis is administered. Due to the devastating risks involved, CPR should be prolonged to provide ample time for the treatment to take effect [6]. The guidelines outlined by the European Resuscitation Council (ERC) [4] suggests at least 60–90 min of continued CPR before considering termination. Although the optimal duration of CPR for CA is still controversial.

A retrospective analysis revealed that universal termination at 20 min of resuscitation could identify over 99% of survivors [7]. In our previous retrospective study, the median CPR duration in patients with sustained ROSC was 8.00 (3.00–15.00) minutes [8]. Subjects with bystander CPR were reported more likely to survive prolonged resuscitation [9] and cases of prolonged resuscitation up to 90 min were also reported in patients with PE-related CA [10, 11]. A CPR duration of 74 min in this case falls within the recommended range of 60–90 min [4]. This successful outcome further supports the validity of the recommended time frame outlined by the ERC [4].

## Prompt administration of thrombolysis

In case 2, the diagnosis of pulmonary embolism was finally confirmed by CTPA examination 2 h after CA. Only then, thrombolysis treatment was initiated. It was later confirmed that haemorrhagic transformation occurred after cerebral infarction. To the best of our knowledge, cerebral infarction is not a typical complication of thrombolysis. The cerebral infarction might be caused by CA, but it was not assumed as a result of thrombolysis.

Once pulmonary embolism is diagnosed intra-arrest, it is recommended to rapidly administer thrombolytic therapy to avoid further complications and missing treatment opportunities. Even when examination and diagnosis are established late, or after a very long CPR duration, it is still appropriate to initiate thrombolytic treatment as soon as possible. Reported cases show successful thrombolytic treatments administered even after 100 to 150 min of CPR [6, 12], demonstrating positive outcomes even when rescue thrombolysis was initiated following extended CPR durations.

It is difficult to diagnose PE intra-arrest, but it is critical to diagnose PE promptly to achieve a higher chance of patient recovery with a response to immediate intervention. The challenge now is how to quickly diagnose pulmonary embolism without extensive testing to ensure uninterrupted management of the patient. Pulmonary embolism was suspected based on the patient’s sedentary lifestyle, refractory pulseless electrical activity, elevated serum D-dimer, and markedly enlarged heart chambers identified by bedside echocardiography. A screening, point-of-care beside ultrasonography may aid help to diagnose PE intra-arrest. In this case, bedside echocardiography or emergency CTPA is recommended for diagnosis [13]. However, ultrasonographic findings should be interpreted with caution in CA induced by PE [14]. In fact, the management of PE is challenging and needs multidisciplinary cooperation.The pulmonary embolism response team (PERT) concept has evolved to facilitate the management. PERT team is composed of clinicians from critical care, interventional radiology, cardiology, cardiothoracic surgery and other specialties [15]. Increasing evidence indicates that PERT implementation improve the treatment and lead to shorter in-hospital stay [16, 17].

### Ruling out cerebrovascular events

In fact, to rule out neurologic contraindications of thrombolysis is as vital as confirming the diagnosis of PE. Tests and physical examination might help identify potential contraindications to thrombolysis. Brain CT is necessary in order to rule out cerebrovascular events such as intracerebral haemorrhage or cerebral infarction. Meanwhile, evidence showed that pupillary abnormalities, characterized by varying pupil size difference of ≥ 2 mm diameter or a dilated pupil of ≥ 5 mm unresponsive to light, independently predicted intracranial haemorrhage, whereas the Babinski reflex and alkalaemia independently predict acute ischemic stroke [18]. For case 3, a negative pupillary light reflex and a pupil diameter of 4 mm were displayed by the patient. Anisocoria can be present in many conditions, such as hypoglycemia, drug overdose, or cerebral hernia. He had no history of drug overdose and his fasting blood sugar level was normal, so our primary suspicion was cerebral hernia. The cerebrovascular event was confirmed by the images revealing presence of subarachnoid haemorrhage. Although, Babinski sign has low sensitivity (50.8%) in identifying pyramidal tract dysfunction, its specificity is almost perfect (99%) [19]. Moreover, abnormal pupils and Babinski signs are late manifestations of cerebrovascular accident. Therefore, expediting an intracranial haemorrhage or cerebral infarction diagnosis may prove to be challenging.

Thrombolysis in patients with CA secondary to PE is not routinely recommended but rely on timing and available expertise [20]. In patients with confirmed PE without contraindication, thrombolysis is a reasonable emergency treatment option. Otherwise, thrombolysis should be judiciously considered after ruling out the contraindications in resuscitation. In case 3, PE was strongly suspected, though thrombolytic treatment was not applied even when ROSC was obtained because the diagnosis was not clear. The CTPA later confirmed massive pulmonary embolism and brain CT scan indicated a complication of subarachnoid haemorrhage. As subarachnoid haemorrhage is definitely a contraindication to thrombolysis. Neither thrombolytic treatment nor anticoagulation was given considering the associated bleeding risk. The patient’s condition continued deteriorating and eventually died.

However, despite the presence of contraindications, thrombolysis should be performed, especially when they are considered as “relative contraindications” [21]. Mercer et al. recommend that even if contraindications are determined, physicians may still consider the possibility of administering thrombolytic therapy, taking into account the risks involved [5]. The careful balance between thrombolysis and bleeding complications in PE treatment is crucial and challenging.

High risk PE with contraindications for thrombolysis or failed thrombolysis can be treated with catheter-directed treatment or surgical embolectomy [22]. These novel treatments offer an important solution for patients with contraindications for thrombolysis with promising results. For example, improvement in right ventricle function and lung perfusion were observed after catheter-directed thrombolysis in two prospective cohort studies [23, 24]. However, most knowledge about catheter-based embolectomy is derived from registries or pooled results from case series [25]. Meanwhile, the technical threshold of these treatments is high, which make these novel treatments difficult to be widely applied in arrest setting.

## Conclusions

The choice of thrombolysis should be made after cautiously ruling out the cerebrovascular events. When the benefit of thrombolysis during CPR outweighs the risk of bleeding, thrombolysis should be given as soon as possible. In addition, patients may benefit from prolonged resuscitation after thrombolysis in PE-related CA.

## Data Availability

All data generated or analysed during this study are included in this published article.

## Abbreviations

CA: Cardiac Arrest
CPR: Cardiac Pulmonary Resuscitation
CT: Computed Tomography
CTPA: Computed Tomography Pulmonary Angiogram
ERC: European Resuscitation Council
ICU: Intensive Care Unit
PE: Pulmonary Embolism
PERT: Pulmonary Embolism Response Team
ROSC: Return of Spontaneous Circulation
rtPA: Recombinant Tissue-type Plasminogen Activator
